# Patterns of cytonuclear discordance and divergence between subspecies of the scarlet macaw (*Ara macao*) in Central America

**DOI:** 10.1007/s10709-023-00193-x

**Published:** 2023-08-23

**Authors:** Matthew L. Aardema, Kari L. Schmidt, George Amato

**Affiliations:** 1 Department of Biology, Montclair State University, Montclair, NJ 07043 USA; 2 Institute for Comparative Genomics, American Museum of Natural History, New York, NY 10024 USA

**Keywords:** Psittacidae, Psittaciformes, Genomic incongruence, Incomplete lineage sorting, Phylogeography, *A. militaris*, *A. chloropterus*

## Abstract

The scarlet macaw, *Ara macao*, is a neotropical parrot that contains two described subspecies with broadly discrete geographical distributions. One subspecies, *A. m. macao*, is found from South America north into southwestern Costa Rica, while the second subspecies, *A. m. cyanoptera*, is found from eastern Costa Rica north into central Mexico. Our previous research using mitochondrial data to examine phylogeographical divergence across the collective range of these two subspecies concluded that they represent distinct evolutionary entities, with minimal contemporary hybridization between them. Here we further examine phylogenetic relationships and patterns of genetic variation between these two subspecies using a dataset of genetic markers derived from their nuclear genomes. Our analyses show clear nuclear divergence between *A. m. macao* and *A. m. cyanoptera* in Central America. Collectively however, samples from this region appear genetically more similar to one another than they do to the examined South American (Brazilian) *A. m. macao* sample. This observation contradicts our previous assessments based on mitochondrial DNA analyses that *A. m. macao* in Central and South America represent a single phylogeographical group that is evolutionarily distinct from Central American *A. m. cyanoptera.* Nonetheless, in agreement with our previous findings, ongoing genetic exchange between the two subspecies appears limited. Rather, our analyses indicate that incomplete lineage sorting is the best supported explanation for cytonuclear discordance within these parrots. High-altitude regions in Central America may act as a reproductive barrier, limiting contemporary hybridization between *A. m. macao* and *A. m. cyanoptera*. The phylogeographic complexities of scarlet macaw taxa in this region highlight the need for additional evolutionary examinations of these populations.

**Supplementary Information:**

The online version contains supplementary material available at 10.1007/s10709-023-00193-x.

## Introduction

The utilization of genetic variation drawn from across the nuclear genomes of many samples has provided new and important insights to the field of avian phylogenetics (e.g., Nadachowska-Brzyska et al. 2013, Prum et al. 2015, Brown et al. 2022). Most importantly, genome-wide datasets have facilitated a more accurate picture of taxonomic relationships than was possible with morphological traits or mitochondrial sequences alone (Kraus and Wink 2015; Toews et al. 2016). The use of mitochondrial markers in avian phylogenetics has proven particularly fraught as several studies have revealed extensive cytonuclear incongruence among related bird species (e.g., Jacobsen and Omland 2011, Dong et al. 2014, Kimball et al. 2021). Accordingly, an understanding of taxonomic relationships that have been established based on physical characteristics and/or mitochondrial analysis alone may be improved by the application of additional, nuclear data (Morin et al. 2004).

The scarlet macaw, *Ara macao*, is a neotropical parrot historically found from southern Mexico through Central America south into the lowland rainforests of South America (BirdLife International 2016). Within *A. macao*, two contemporary subspecies have been described: *A. m. macao* and *A. m. cyanoptera* (Wiedenfeld 1994). *A. m. macao* is reported to be relatively smaller and typically has some green coloration on its secondary wing coverts, while *A. m. cyanoptera* is generally larger, and has very little to no green on its wings (Figure S1). These two subspecies are broadly allopatric with *A. m. cyanoptera* being found only in Central America from southern Mexico to northeast Costa Rica, and *A. m. macao* being found from western Costa Rica south into South America. An intraspecific hybrid zone is proposed to exist in northern Costa Rica and possibly southern Nicaragua based on the presence of birds with coloration appearing intermediate between the two subspecies (Wiedenfeld 1994). However, our recent analysis comparing mitochondrial data from across the range of scarlet macaws found no populations in which haplotypes of both subspecies were present (Schmidt et al. 2020). Rather, *A. m. cyanoptera* haplotypes were only found on the eastern side of the Costa Rican Cordillera Central (Figure S1), while *A. m. macao* haplotypes were only found on the western side of these same higher elevation areas. Based on this result, we proposed that these central highlands act as a barrier to genetic exchange between the two subspecies (Schmidt et al. 2020). In this same study, we also proposed that *A. m. cyanoptera* and *A. m. macao* represent two distinct, monophyletic groups, with *A. m. macao* found in Central America being more closely aligned evolutionarily with South American *A. m. macao* than with Central American *A. m. cyanoptera*.

Here we expand on this previous work by using a large number of genetic markers drawn from across the nuclear genomes of multiple *A. macao* samples to further examine the phylogenetic relationships of these birds in Central America. We explicitly test the hypothesis that the two subspecies found in this region are genetically distinct and reproductively isolated. In light of our findings, we also investigate patterns of phylogenetic discordance, comparing the relative roles of incomplete linage sorting and post-divergence hybridization in generating cytonuclear divergence between the examined *A. macao* populations.

## Materials and methods

### Samples, DNA extraction, and genome sequencing

To develop a large number of nuclear genetic markers for our phylogenetic investigations, we utilized next-generation sequencing data (Illumina). At the time of our study, the number of *Ara* genomes publicly available was limited, so we were required to generate our own datasets. To do this, we selected samples of both *A. macao* subspecies that were obtained from Central America. Two samples came from Laguna del Tigre National Park in Guatemala, and an additional four samples were from Costa Rica. These Costa Rican samples were captive parrots that were part of the Ara Project (https://macawrecoverynetwork.org/). Although these birds were assumed to derive from Costa Rica, the specific source locations were unknown as these parrots were either confiscated birds or pets that had been donated by their owners. Subspecific taxonomic designations for all six samples were previously determined based on microsatellite analysis (Hains 2015). The two Guatemalan samples were classified as *A. m. cyanoptera*, as was one of the Costa Rican samples (Table 1, Table S1). The other three Costa Rican samples were classified as *A. a. macao.* For outgroup comparisons, we also sequenced a single sample of the red-and-green macaw, *A. chloropterus*.

**Table 1:** Sample information, taxonomic designation, country of origin, sex, and relative diversity estimate (π) for all samples used in this study. The Guatemalan samples were both from Laguna del Tigre National Park. The Costa Rican samples were captive parrots whose specific source locations were unknown (see text for more details)

Sample ID	Taxon	Country of Origin	Sex	Relative Diversity (π; 2,571 sites)	Genome Reference
**AmilUK06**	*Ara militaris*	Unreported	Unknown	0.1101	Hains et al. 2020
**AchlUK02**	*A. chloropterus*	Unknown	Male	0.1287	This Study
**AchlUK08**	*A. chloropterus*	Unreported	Unknown	0.1155	Hains et al. 2020
**AmmaBR01**	*A. m. macao*	Brazil	Female	0.1548	Seabury et al. 2013
**AmmaCR01**	*A. m. macao*	Costa Rica	Male	0.1741	This Study
**AmmaCR02**	*A. m. macao*	Costa Rica	Male	0.1787	This Study
**AmmaCR16**	*A. m. macao*	Costa Rica	Female	0.1706	This Study
**AmcyCR10**	*A. m. cyanoptera*	Costa Rica	Male	0.1591	This Study
**AmcyGT02**	*A. m. cyanoptera*	Guatemala	Female	0.1653	This Study
**AmcyGT04**	*A. m. cyanoptera*	Guatemala	Male	0.1661	This Study

From each sample, we extracted DNA from whole blood using the DNeasy Blood & Tissue Kit (QIAGEN Inc.), following the manufacturer’s recommended protocol. Next, we sheared this extracted DNA with a Covaris ultrasonicator (Covaris, Woburn, MA). With the resulting fragments, we constructed standard 2 × 150 nucleotide libraries with barcoded adapters using the Illumina TruSeq Library Preparation kit following the standard protocol (Illumina, San Diego, CA). After library preparation we combined the barcoded samples in two separate pools and these multiplexed libraries were sequenced on an Illumina HiSeq X Ten at the New York Genome Center (NYGC).

### Read mapping and variant calling

To our seven previously unpublished Illumina read sets (see above), we added comparable published data (raw Illumina reads) from a single Brazilian sample of *A. m. macao* (Seabury et al. 2013), as well as one sample each of the outgroups *A. militaris* and *A. chloropterus* (Hains et al. 2020). Independently for each of these 10 samples’ read data, we used the program Trim Galore (https://github.com/FelixKrueger/TrimGalore) to trim bases from read ends with a quality score (Q score) less than 20. Any subsequent read pair for which either read was less than 30 nucleotides long was then removed from the dataset. Next, we used the program BWA v.0.7.12 (Li 2013) with the ‘MEM’ algorithm to map our trimmed reads to the *Ara macao* reference genome, v.1.1 (GenBank Accession: GCA_000400695.1; Seabury et al. 2013). Following read mapping, we used the tool ‘AddOrReplaceReadGroups’ within the Picard toolkit v.1.119 (http://broadinstitute.github.io/picard/) to add read groups and sort the mapped reads. We then identified and marked read duplicates using the Picard tool ‘MarkDuplicates’. We realigned indels using the ‘IndelRealigner’ tool in the Genome Analysis Toolkit (‘GATK’) v.3.8.1 (McKenna et al. 2010), then for each sample, we called variant sites using GATK’s ‘HaplotypeCaller’ (specific flags: --emitRefConfidence GVCF, --variant_index_type LINEAR, --variant_index_parameter 128000 -rf BadCigar).

Once all samples were processed, we collectively genotyped them using GATK’s ‘GenotypeGVCFs’ tool, producing one multi-sample variant call format (VCF) file with all samples and identified (‘called’) genetic variants. We used GATK’s ‘SelectVariants’ tool to limit our dataset to just single nucleotide polymorphisms (SNPs), then used this same tool to remove variants with a quality by depth less than 6 (QD < 6.0), Fisher strand bias greater than 40 (FS > 40.0), mapping quality less than 59 (MQ < 59.0), mapping quality rank sum less than − 0.3 (MQRankSum < -0.3), read position rank sum less than − 2 (ReadPosRankSum < -2.0), and a strand odds ratio greater than 2 (SOR > 2.0). These filtering thresholds were determined based on the observed variant distributions for these parameters (Figure S2), and all were equal to, or more stringent than, the developer’s recommended cutoffs (DePristo et al. 2011).

### Reference genome annotation

To categorize the segregating variants in our SNP dataset (e.g., ‘intronic’, ‘missense’, ‘non-synonymous’, etc.), we produced gene predictions for the *Ara macao* reference genome using the program BRAKER2 v.2.1.5 (Brůna et al. 2021), incorporating AUGUSTUS v.3.4.0 (Stanke et al. 2006). The program GenomeThreader v.1.7.1 (Gremme et al. 2005) was used within BRAKER2 to generate training gene structures based on protein sequences from the annotated parrot (Psittaciformes) *Melopsittacus undulatus* (“budgerigar”, GenBank Accession: GCA_012275295.1). We then annotated our filtered SNP VCF file using the program SnpEff v.4.3 (Cingolani et al. 2012), with a custom annotation database built using the *A. macao* reference genome and the general feature format (GFF) file generated with BRAKER2.

### Phylogenetic analyses

We conducted two phylogenetic analyses, one utilizing genetic markers from the nuclear genome and the other using assembled, whole mitochondrial genomes. To establish our nuclear genetic markers, we first removed mitochondrial regions from our VCF dataset using the ‘SelectVariants’ tool in GATK v.4.2.5. We then used the ‘VariantFiltration’ and ‘SelectVariants’ tools to designate any variant within a sample with a depth of coverage less than 8× as ‘no call’. Next, we used the ‘SelectVariants’ tool to retain only genetic variants for which all samples were genotyped (flag: --max-nocall-number 0). We then used the program bcftools v.1.15 (Li 2011) to select all 4-fold synonymous (‘silent’), segregating sites as annotated in the *A. macao* reference genome (see above). Our choice to use only 4-fold synonymous sites was made to minimize variance in divergence rates across sites (Wright and Andolfatto 2008), and allow for the effective application of a single mutation model. For variants present on the same scaffold, we used a sliding window of 50 SNPs at 10 SNP increments between windows to thin SNPs if their pairwise squared correlation (r^2^) was greater than 50% (Novembre et al. 2008). This was done using PLINK v.1.90b6.6 (Purcell et al. 2007). Our final VCF for nuclear phylogenetic analysis consisted of 8,443 unlinked, 4-fold synonymous genetic markers.

We converted this filtered VCF to PHYLIP format using the vcf2phylip.py v.1.5 python script (https://zenodo.org/record/1257058#.YJL3ymZKi6t). We then used jModelTest v.2.1.10 (Guindon and Gascuel 2003; Darriba et al. 2012) with default settings to select the best-fit model of nucleotide substitution for this dataset based on AIC score. The model that was selected was the transversion model (‘TVM’, Posada 2003). Using this model, we carried out a maximum-likelihood phylogenetic analysis with PhyML v.3.1 (Guindon et al. 2010), applying 100 non-parametric bootstrap replicates to determine confidence values for the observed relationships between samples. The resulting phylogenetic tree was visualized with FigTree v.1.4.4 (Rambaut 2018).

For the mitochondrial phylogenetic analysis, we used the ‘FastaAlternateReferenceMaker’ tool in GATK along with our quality filtered VCF file (see above) to produce mitochondrial genomes for each sample (reference scaffold: CM002021.1). These mitochondrial genomes were then combined into a single, aligned FASTA file. To determine the best partitioning scheme and nucleotide substitution model for this data we used PartitionFinder v.2.1.1 (Lanfear et al. 2016), considering all models. The first, second, and third positions for each of the 13 mitochondrial coding regions were examined separately, and the small-sample size corrected version of the Akaike Information Criterion (AICc) was used to select the best partitioning scheme. With this scheme (Table S2), we conducted a maximum-likelihood (ML) phylogenetic analysis using IQtree v.1.6.12 (Nguyen et al. 2015). To determine support for each node we generated 1000 ultrafast bootstrap approximation (UFBoot) replicates (Hoang et al. 2018).

### Sample clustering and admixture

To examine sample clustering, we retained likely ‘neutral’ sites from our filtered VCF dataset (sample-SNP read depth ≥ 8× and no missing data across all samples; see above). We defined neutral sites as those annotated by us in the *A. macao* genome as ‘intronic’ or ‘synonymous’. We used only likely neutral sites to reduce the possibility that past differential selection acting on protein structure or gene expression would obscure historical phylogeographic patterns (Wright and Andolfatto 2008).

After filtering to retain only likely neutral sites, we removed the *A. militaris* outgroup sample, along with any resulting non-segregating sites. We then filtered the remaining sites for linkage as described above. This left us with 59,028 segregating, neutral, genetic markers. With these markers, we used a principal component analysis (PCA) to investigate clustering among the *A. chloropterus* and *A. macao* samples. This PCA was carried out with the program PLINK v.1.90b6.6 (Purcell et al. 2007), and the results were visualized using R v.4.0.2 (R Core Team 2020).

We also wanted to examine sample clustering utilizing just the *A. macao* samples. From our VCF file of likely neutral SNPs, we removed the three outgroup samples (one *A. militaris* and two *A. chloropterus*), and subsequently any variants that were no longer segregating. We then filtered for linkage as described above. This left us with 43,487 neutral, segregating genetic markers that we then used to perform a second PCA.

We also used this dataset of 43,487 neutral, segregating genetic markers to examine population structure and ancestry proportions among our *A. macao* samples with a maximum likelihood approach. This was done using the program ADMIXTURE v.1.3.0 (Alexander et al. 2009; Alexander and Lange 2011). For this analysis, we examined potential sample clusters (K) from one to five. Each K value was run 20 independent times with different seed values used for each run. Across K values, means observed for the standard error of the 10-fold cross-validation (CV) error estimate were compared to identify the best supported number of clusters represented in the data. Smaller mean CV values support greater confidence in the number of clusters modeled (Alexander et al. 2015). We used the online version of CLUMPAK (http://clumpak.tau.ac.il/, Kopelman et al. 2015), with default settings to determine the mean q-matrix cluster assignment for each sample, at each K value.

### Genetic diversity and genome-wide divergence

To examine relative genetic diversity within each sample/population as well as divergence between samples and populations, we first generated a nuclear genetic dataset using the ‘VariantFiltration’ and ‘SelectVariants’ tools to designate any variant within a sample with a depth of coverage less than 15× as ‘no call’. This more stringent filter for read depth relative to the earlier analyses described was done to improve confidence in the called homozygous/heterozygous state for each site within each sample. Read depths of at least 15× have been shown to be sufficient to accurately genotype segregating sites in genomic data with greater than 98% confidence (Song et al. 2016).

After depth filtering, we used the ‘SelectVariants’ tool in GATK to retain only SNPs for which all samples were genotyped. We also used the ‘SelectVariants’ tool to retain only biallelic variants, then selected only likely ‘neutral’ sites for the reasons described above (annotated as ‘intronic’ or ‘synonymous’). After filtering, we retained 2,571 biallelic, neutral, segregating sites. We used PLINK v.1.90b6.6 to convert each sample’s genotype at each site to a numeric value (0 or 2 = homozygous; 1 = heterozygous), using the ‘-recodeA’ function. The resulting file was manually edited to remove the header and the excess columns generated by PLINK (e.g., population, sex, phenotype, etc.). We then used a custom Perl script to determine nucleotide diversity (π) for each sample individually at each site (Formula 10.5, Nei 1987). The mean π value was calculated across all examined sites. Although these mean values were not absolute measures of genome-wide diversity for these samples, they did allow for relative comparisons between samples, populations, and taxa. We also assessed nucleotide differentiation between pairs of samples (d_XY_) for each site (Eq. 10.20, Nei 1987), as well as the net number of nucleotide substitutions per site after accounting for within sample π (d_A_; Eq. 10.21; Nei 1987). Means for these metrics (d_XY_ & d_A_) were then calculated for each pairwise-sample comparison across all examined sites.

### Patterns of incomplete lineage sorting and introgression

We first wanted to assess the extent of phylogenetic incongruence between South American *A. m. macao*, and each of the two Central American *A. macao* subspecies. Based on our previous findings (Schmidt et al. 2020), we postulated greater rates of post-divergence genetic exchange between South and Central American *A. m. macao*, relative to South American *A. m. macao* and Central American *A. m. cyanoptera.* To assess this hypothesis, we utilized the ABBA/BABA test to calculate Patterson’s D statistic (Green et al. 2010), using the dataset of 59,028 segregating, neutral genetic markers as in our interspecific clustering analysis (*A. militaris* excluded; see above). The D statistic is determined by comparing shared genetic variation between three focal taxa or populations and an outgroup, and determining whether phylogenetically informative sites agree with the primary phylogeny (‘AABB’ sites), or support one of the two possible alternative relationships (‘ABBA’ or ‘BABA’ sites). We used the default parameters of the ‘Dtrios’ option within the program Dsuite v.0.4r38 to estimate the relative frequencies of AABB, ABBA, and BABA sites (Malinsky et al. 2021). From these, we also used Dsuite to calculate the D statistic. A D statistic that statistically deviates from zero (‘0’) suggests genetic exchange after divergence (‘introgression’), whereas a D statistic that does not deviate from zero supports incomplete linage sorting (ILS) as the primary cause of phylogenetic incongruence. Dsuite was used to determine statistical significance via a jackknife approach, subsampling blocks of variants. A result was considered significant at p ≤ 0.05. For this analysis, the two *A. chloropterus* samples were used as the outgroup, and we compared genetic variation between the Brazilian *A. m. macao* sample (P3), the *A. m. macao* samples from Costa Rica (P2), and the *A. m. cyanoptera* samples from both Costa Rica and Guatemala combined (P1). Because variation in geographic distance could affect patterns of introgression (i.e., Guatemala is geographically further away from South America than is Costa Rica), we also performed a second analysis, with only the Costa Rican *A. m. cyanoptera* sample (Guatemalan *A. m. cyanoptera* excluded).

We additionally wanted to examine the extent of possible genetic exchange between the two subspecies in Central America. Here we postulated that if post-divergence genetic exchange has occurred between Central American *A. m. macao* and *A. m. cyanoptera* populations, then the Central American *A. m. macao* samples (all from Costa Rica) would share more alleles with the *A. m. cyanoptera* from Costa Rica then with the *A. m. cyanoptera* from Guatemala. This analysis was conducted using the same methodologies as previously described, but with the *A. macao* only dataset of 43,487 neutral, segregating genetic markers (see above).

## Results

### Phylogenetic analyses

**Fig. 1:**
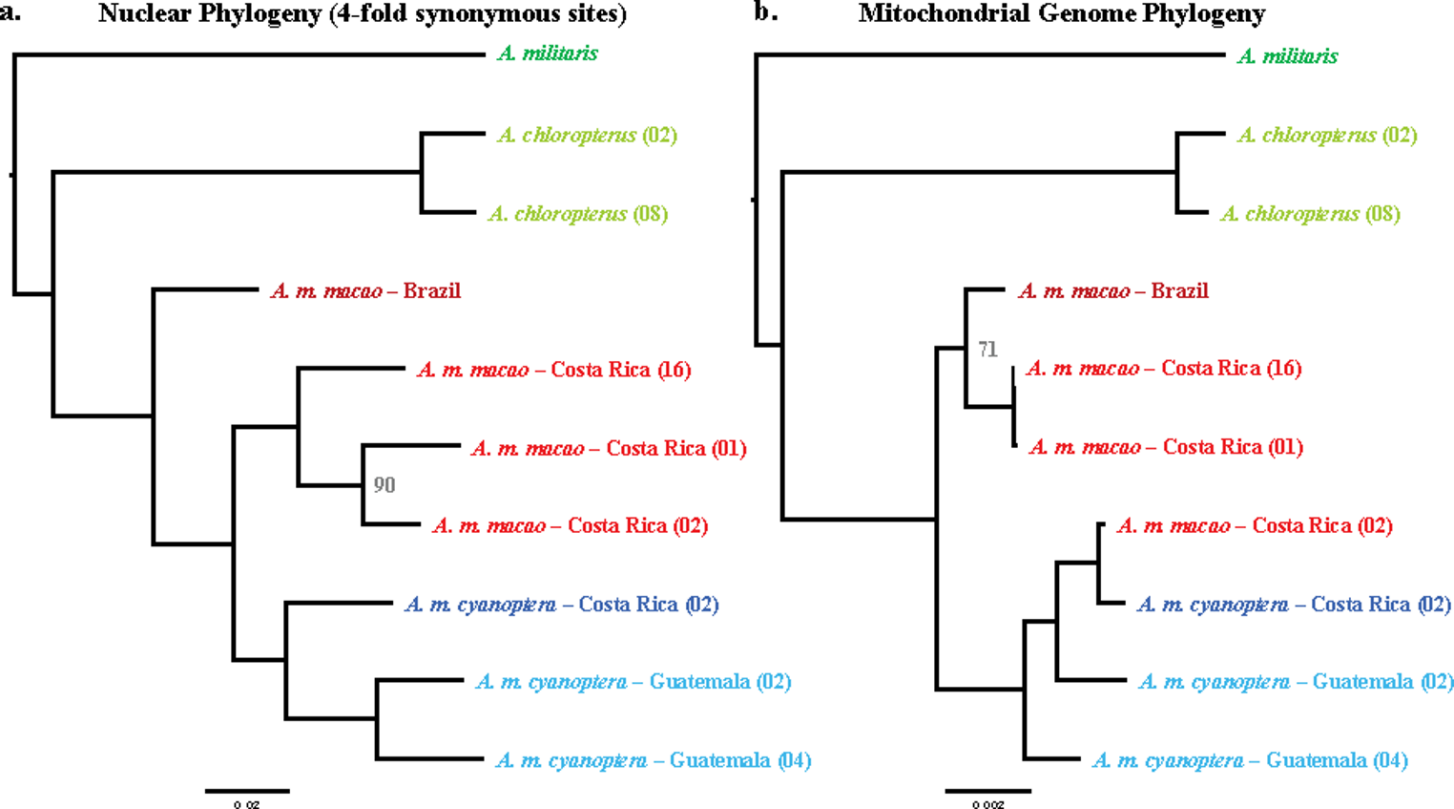
Maximum likelihood phylogenic analyses with (a) 4-fold synonymous nuclear SNPs, and (b) mitochondrial genomes. The colors for the branch tip labels correspond to the different species, subspecies, and geographic regions of samples examined in this study. Where less than 100, the numbers at branch nodes indicate bootstrap support for each bifurcation in the tree (out of 100)

Considering our nuclear phylogeny, *A. militaris* was the most evolutionary distinct sample examined (Fig. 1a). The two *A. chloropterus* samples were clustered with one another and formed a sister clade to the *A. macao* samples. Within *A. macao*, the Brazilian *A. m. macao* sample was sister to all the Central American samples. The Central American *A. macao* samples from both subspecies each formed a monophyletic clade, and these were sister to one another. Within *A. m. cyanoptera*, the two Guatemalan samples of *A. m. cyanoptera* clustered together, apart from the Costa Rican sample. All nodes had 100/100 bootstrap support except for the node joining the Costa Rican *A. m. macao* samples (90/100).

In our phylogenetic analysis using whole mitochondrial genomes, the interspecific relationships were identical to those observed with our nuclear data. *A. militaris* was the most diverged taxon, and sister to a clade formed by the *A. chloropterus* and *A. macao* samples (Fig. 1b). However, among the *A. macao* samples there were several relationships that differed from those inferred from the nuclear data. First, the *A. m. macao* sample from Brazil clustered with two of the three *A. m. macao* samples from Costa Rica. These three samples formed a clade that was sister to a second *A. macao* clade formed by the remaining four samples. Within this clade were the three samples of *A. m. cyanoptera* and one sample of *A. m. macao*. This *A. m. macao* was from Costa Rica and clustered with the single *A. m. cyanoptera* sample from Costa Rica. All nodes had 100/100 bootstrap support except for the node joining two of the Costa Rican *A. m. macao* samples and the Brazilian *A. m. macao* sample (71/100).

### Sample clustering

**Fig. 2:**
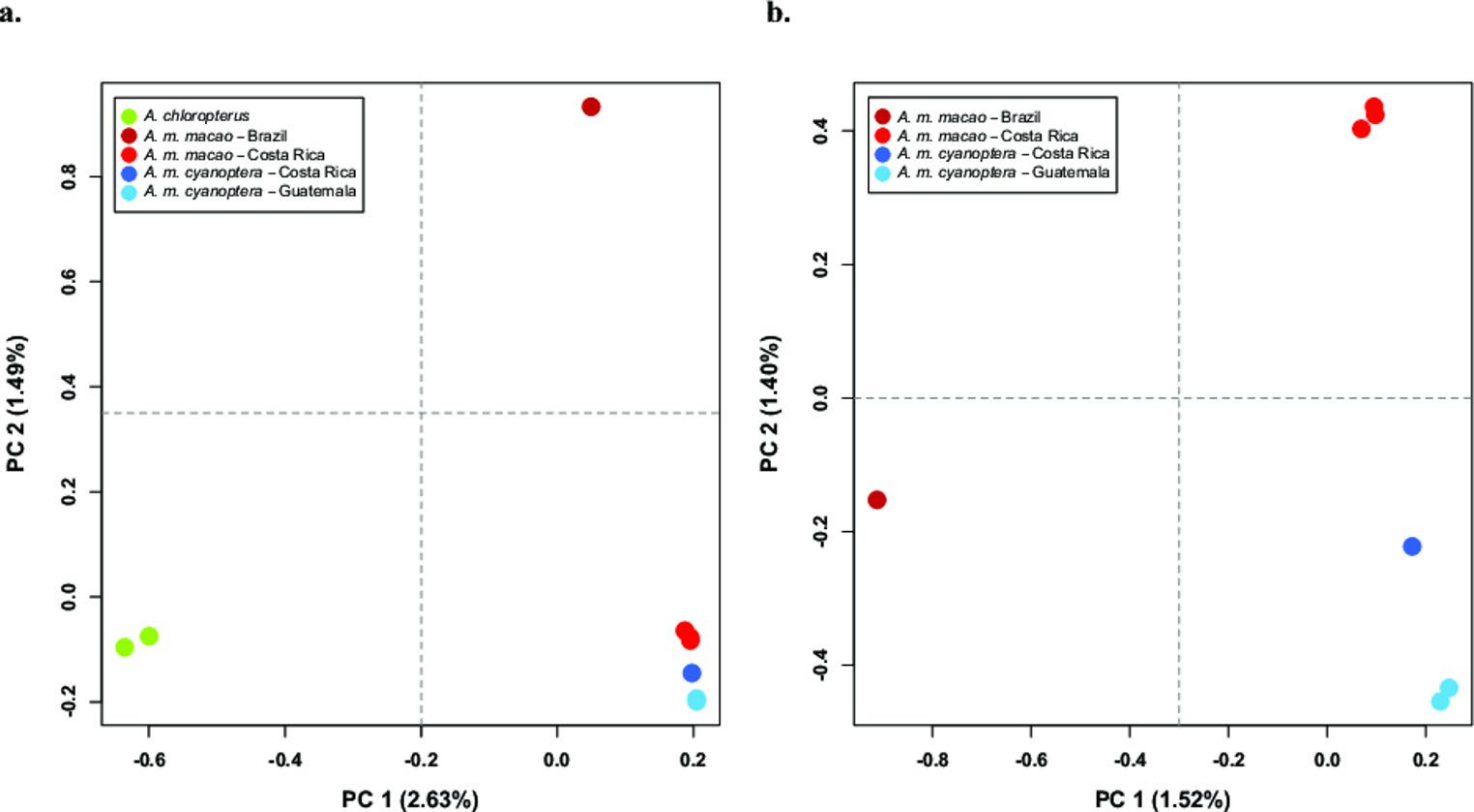
Principal component analyses (PCAs) based on the filtered intergenic SNPs dataset. (a) Analysis included the seven samples of *A. macao* as well as the two samples of *A. chloropterus.* (b) Only *A. macao* samples included in the analysis. These PCAs were implemented in PLINK and plotted with R; the first two PCs are shown. The percentages in the parentheses along each axis indicate the amount of genetic variation captured by the principal component. Colors correspond to the different species, subspecies, and geographic areas, and are consistent between the two PCAs.

Our principal component analysis that included the two *A. chloropterus* showed the greatest differentiation between this species and the collective *A. macao* samples along PC 1 (Fig. 2a). This principal component captured 2.63% of the observed genetic variance among the samples. Along PC 2, all the Central American samples formed a tight cluster, and the Brazilian *A. m. macao* sample was distinct. This principal component accounted for 1.49% of the observed genetic variance. In our PCA with only *A. macao* samples, PC 1 distinguished the Brazilian *A. m. macao* sample from the Central American samples, and captured 1.52% of the observed genetic variance among samples (Fig. 2b). Along PC 2, the Costa Rican *A. m. macao* samples formed a distinct cluster from the *A. m. cyanoptera* samples. The single *A. m. cyanoptera* from Costa Rica was slightly separated from the two *A. m. cyanoptera* samples from Guatemala, being shifted primarily along PC 2 towards the Costa Rican *A. m. macao* samples. PC 2 accounted for 1.40% of the observed genetic variance among these *A. macao* samples.

**Fig. 3:**
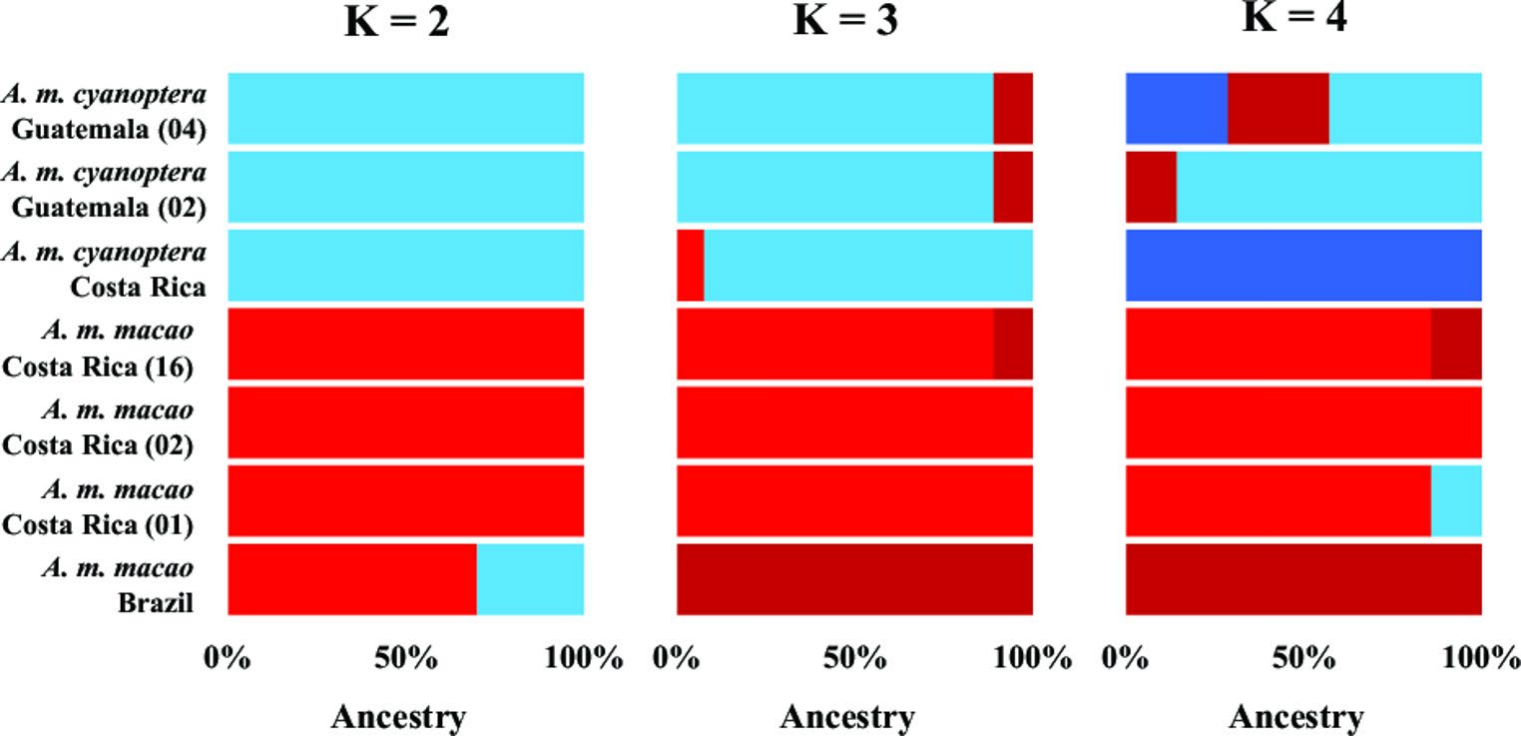
Bar plots showing relative proportions of genetic ancestry for the *A. macao* samples plotted for genetic clusters (K) from two to four. For each horizontal bar, the relative proportions of each color indicate the proportion of genetic ancestry from each cluster assigned to that sample. Sample designations are reported along the vertical axis. Blue colors correspond to *A. m. cyanoptera* ancestry, while red colors correspond to *A. m. macao* ancestry

In our ADMIXTURE analysis, the lowest mean cross-validation (CV) error across K values 1–5 was for one (1.306, SE = 0.001, Table S3). At K = 2 the Central American *A. m. macao* samples were clearly distinguished from the *A. m. cyanoptera* samples (Fig. 3, Table S4), with no evidence of mixed ancestry. Interestingly, the *A. m. macao* sample from Brazil did harbor a mixture of ancestry with 70% of its genetic background being most aligned with the *A. m. macao* from Central America, and 30% of its genetic background being more aligned with the *A. m. cyanoptera* samples from Central America. At K = 3, the Brazilian *A. m. macao* sample was distinct, with ancestry matching it being found in the two *A. m. cyanoptera* samples from Guatemala and one Costa Rican *A. m. macao.* The *A. m. cyanoptera* sample from Costa Rica also had a small amount of ancestry (7.5%) that matched the *A. m. macao* samples from Costa Rica. At K = 4, the Costa Rican *A. m. cyanoptera* sample became distinct from the two Costa Rican *A. m. cyanoptera* samples. Several samples also showed mixed ancestry at K = 4.

### Diversity and divergence

Across the samples/taxa examined here, the *A. militaris* sample had the lowest estimated relative genetic diversity with a calculated π of 0.1101 (Table 1). The *A. chloropterus* samples had a mean π of 0.1221 (SD: 0.0094). Among *A. m. macao*, the sample from Brazil had the lowest calculated diversity with a π calculation of 0.1548. The *A. m. macao* samples from Costa Rica had the highest calculated relative diversity with a mean π of 0.1745 (SD: 0.0041). The *A. m. cyanoptera* samples from Costa Rica and Guatemala had a slightly lower mean π calculation of 0.1635 (SD: 0.0038).

In our examination of population differentiation, the highest mean pairwise d_XY_ value was observed between the *A. militaris* sample and the Costa Rican *A. m. macao* samples (d_XY_=0.2922, SD = 0.0036; Table 2, Table S5). The lowest mean pairwise d_XY_ values was observed between the *A. m. cyanoptera* samples from Guatemala and the *A. m. cyanoptera* sample from Costa Rica (d_XY_=0.2462, SD = 0.0047). Individual pairwise estimates of d_XY_ were lowest for intrapopulation comparisons (Table S5).

**Table 2:** Within taxa/population calculations of genetic differentiation at 2,571 segregating, biallelic, neutral sites (‘intronic’ or ‘synonymous’). Mean divergence (d_XY_) is given above the diagonal axis. Calculations were performed for all pairwise sample comparisons and means (where appropriate) determined from these values (see Table S5 for individual calculations). Mean divergence after accounting for within sample genetic variation (d_A_; [d_XY_ – π]) is given below the diagonal axis. As with d_XY_, calculations were performed for all pairwise sample comparisons and means (where appropriate) determined from these values (see Table S5 for individual calculations). Standard deviations for all mean estimates are given in parentheses

	*A. militaris* (n = 1)	*A. chloropterus* (n = 2)	*A. m. macao* - Brazil (n = 1)	*A. m. macao* - Costa Rica (n = 3)	*A. m. cyanoptera* - Costa Rica (n = 1)	*A. m. cyanoptera* - Guatemala (n = 2)	
***A. militaris*** **(n = 1)**		0.2584 (0.0100)	0.2686 (na)	0.2922 (0.0036)	0.2874 (na)	0.2899 (0.0034)	**d**_**XY**_
***A. chloropterus*** **(n = 2)**	0.1423 (0.0054)		0.2626 (0.0117)	0.2880 (0.0067)	0.2864 (0.0067)	0.2895 (0.0067)	
***A. m. macao*** **- Brazil (n = 1)**	0.13613 (na)	0.1242 (0.0070)		0.2603 (0.0081)	0.2526 (na)	0.2599 (0.0001)	
***A. m. macao*** **- Costa Rica (n = 3)**	0.1500 (0.0035)	0.1397 (0.0039)	0.0957 (0.0062)		0.2538 (0.0042)	0.2576 (0.0047)	
***A. m. cyanoptera*** **- Costa Rica (n = 1)**	0.1529 (na)	0.1458 (0.0021)	0.0957 (na)	0.0870 (0.0032)		0.2462 (0.0047)	
***A. m. cyanoptera*** **- Guatemala (n = 2)**	0.1520 (0.0037)	0.1456 (0.0037)	0.0997 (0.0004)	0.0875 (0.0050)	0.08382 (0.0049)		
	**d**_**A**_						

When we looked at divergence estimates after accounting for within sample diversity (d_A_), the highest mean pairwise d_A_ value was observed between the *A. militaris* sample and the *A. m. cyanoptera* sample from Costa Rica (d_A_=0.1529, Table 2). The lowest mean pairwise d_A_ value was again observed between the Guatemalan *A. m. cyanoptera* samples and the Costa Rican *A. m. cyanoptera* sample (d_A_=0.0838, SD = 0.0049). As with d_XY_, individual pairwise estimates of d_A_ were lowest for intrapopulation comparisons (Table S5).

### Genetic incongruence

**Fig. 4:**
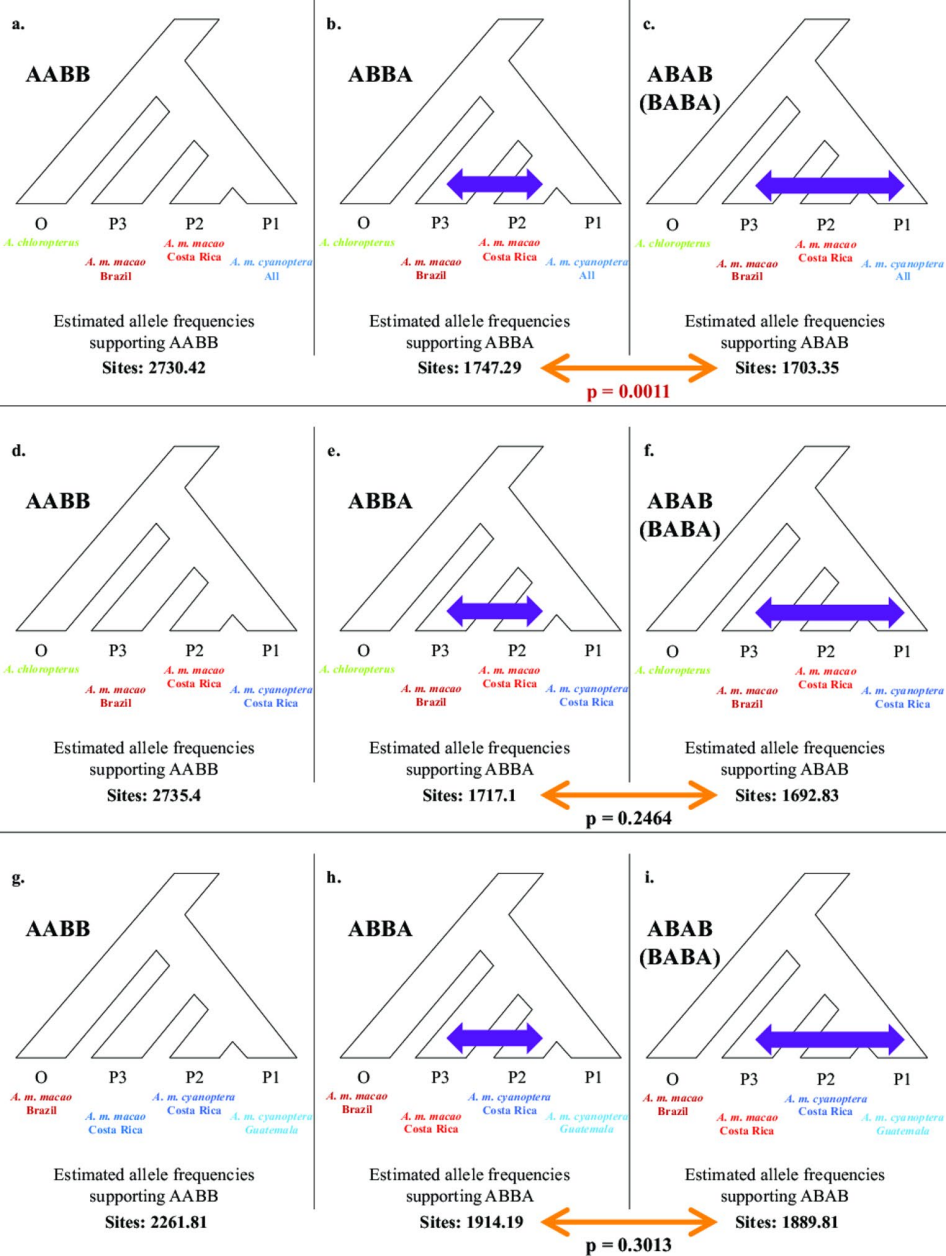
The estimated number of sites supporting the primary phylogenetic relationship (AABB sites), or one of two alternative phylogenetic relationships (ABBA or ABAB, respectively), for four-way taxonomic comparisons (one outgroup and three taxa for comparisons). a-c) Assessment with *A. chloropterus* as the outgroup, and comparing the *A. m. macao* sample from Brazil, the *A. m. macao* samples from Costa Rica, and the *A. m. cyanoptera* samples from Costa Rica and Guatemala combined. d-f) Assessment with *A. chloropterus* as the outgroup, and comparing the *A. m. macao* sample from Brazil, the *A. m. macao* samples from Costa Rica, and only the *A. m. cyanoptera* sample from Costa Rica. g-i) Assessment with the *A. m. macao* sample from Brazil as the outgroup, and comparing the *A. m. macao* samples from Costa Rica, the *A. m. cyanoptera* sample from Costa Rica, and the *A. m. cyanoptera* samples from Guatemala. Only the first comparison (a-c) revealed a statistically significant difference between ABBA and BABA sites (p = 0.0011)

The estimated site frequencies for all ABBA/BABA analyses are given in Fig. 4. Our first examination of phylogenetic incongruence was between the Brazilian *A. m. macao* sample and the Central American *A. m. macao* and *A. m. cyanoptera* samples. With 59,028 segregating, neutral, genetic markers, Dsuite produced 20 jackknife blocks, each with 2,950 variants, for its statistical estimation of genetic incongruence patterns. The resulting D statistic was 0.0127, which was statistically different from 0 (Z = 3.2587, p = 0.0011). Within the data, the number of estimated sites indicating a shared relationship between the Brazilian *A. m. macao* and the Central American *A. m. macao* samples (‘ABBA’) was 1747.29, whereas the number of estimated sites shared between the Brazilian *A. m. macao* and the Central American *A. m. cyanoptera* samples (‘BABA’) was 1703.35. This result suggests genetic exchange has occurred between the Brazilian *A. m. macao* and the Central American *A. m. macao*, after *A. m. macao* and *A. m. cyanoptera* began to diverge. However, when we controlled for differences in geographic distance from Brazil by only using Costa Rican *A. macao* samples (Fig. 4), the degree of estimated incongruence between the Brazilian *A. m. macao* and either subspecies of *A. macao* in Central America was no longer statistically different (D = 0.0071, Z = 1.1590, p = 0.2464).

For our comparison of genetic incongruence between the Costa Rican *A. m. macao* sample, and the samples of *A. m. cyanoptera* from either Costa Rica or Guatemala (Fig. 4), we utilized 43,487 neutral, segregating genetic markers. With these, Dsuite produced 20 Jackknife blocks, each with 2,173 variants. The D statistic from this analysis was 0.0064, which was not statistically different from 0 (Z = 1.0337, p = 0.3013).

## Discussion

We used genetic markers derived from across the genomes of multiple parrots in the macaw genus *Ara* to assess evolutionary relationships within these birds. In particular, we examined phylogenetic associations and patterns of genomic incongruence between the two recognized subspecies of the scarlet macaw, *A. macao*. Our phylogeny based on likely neutral, nuclear genetic variants showed a clear division between the two subspecies in Central America (Fig. 1a). However, collectively these samples formed a single clade that was sister to the Brazilian *A. m. macao* sample. This result contradicts our previous finding based on mitochondrial data that South and Central American *A. m. macao* are more closely aligned to one another than Central American *A. m. macao* is to Central American *A. m. cyanoptera* (Schmidt et al. 2020).

In our phylogeny based on full mitochondrial genomes (Fig. 1b), we observed a more complex pattern of taxonomic relationships. First, the Brazilian *A. m. macao* sample clustered with two of the three *A. m. macao* samples from Costa Rica. This pattern is similar to our previous results using mitochondrial loci sequenced from a broad sampling of South and Central American *A. macao* (Schmidt et al. 2020). However, we also observed that a third Costa Rican *A. m. macao* sample clustered with the three *A. m. cyanoptera* samples. This observation may indicate introgression of the *A. m. cyanoptera* mitochondrial genome into the Costa Rican population of *A. m. macao.* Comparable mitochondrial introgression has been observed previously in birds, even across species lines (e.g., Pons et al. 2014, Andersen et al. 2021).

Our principal component analyses broadly recapitulated the results of our nuclear phylogeny. In our PCA including the outgroup *A. chloropterus*, the greatest differentiation among the *A. macao* samples (along PC 2) was between the Brazilian *A. m. macao*, and the Central American *A. macao* samples (Fig. 2a). When *A. m. chloropterus* was excluded from the analysis, differentiation between the Brazilian *A. m. macao* samples and those from Central America was observed along PC1, and the Central American *A. m. macao* and *A. m. cyanoptera* formed separate clusters along PC 2 (Fig. 2b). The Costa Rican *A. m. cyanoptera* sample was somewhat separate from the Guatemalan *A. m. cyanoptera* samples along PC 2. This observation could indicate potential genetic exchange (‘hybridization’) between *A. m. macao* and *A. m. cyanoptera* in Costa Rica (Scordato 2020, Stephens et al. 2020). Intriguingly, the three Costa Rican *A. m. macao* samples form a tight cluster, with no reciprocal evidence of possible admixture with *A. m. cyanoptera*. This result suggests that the potential introgression indicated by our phylogenetic analyses may be limited to the mitochondrial genome.

In our ADMIXTURE analyses, K = 1 was the most strongly supported value (Table S3). This broadly means that all samples are from the same population with minimal taxonomic differentiation. Nonetheless, in such analyses an examination of genomic admixture at higher K values can still be informative (Liu et al. 2020). At K = 2 we observed a clear distinction between the Central American *A. m. macao* and *A. m. cyanoptera* samples, with no evidence of mixed ancestry (Fig. 3). However, the Brazilian sample contained ancestry associated with both the *A. m. macao* and *A. m. cyanoptera* samples from Central America. This observation may indicate incomplete linage sorting withing the Central American populations during their divergence (Wang et al. 2019). The higher percentage (70%) of observed *A. m. macao* ancestry in the Brazilian sample could also be the result of either ongoing genetic exchange between South and Central American *A. m. macao* (Lavretsky et al. 2019), or else substantial derived genomic divergence in *A. m. cyanoptera* (Dávalos et al. 2012). At K = 3, the Brazilian *A. m. macao* sample had a unique ancestry, and we observed small amounts (< 12%) of admixture in several other samples. At K = 4, the single Costa Rican sample of *A. m. cyanoptera* had a unique ancestry, although one Guatemalan *A. m. cyanoptera* also contained 28.6% of this genetic background. Broadly our ADMIXTURE results are in accordance with predictions based on the prior geographic and subspecies designations.

Although collected from two separate, non-bordering countries, the three *A. m. cyanoptera* samples appeared to harbor less relative genetic diversity (mean π = 0.1635, SD = 0.0038) than did the three Costa Rican *A. m. macao* samples (mean π = 0.1745, SD = 0.0041). This finding may reflect the more restricted geographic range of *A. m. cyanoptera* as well as its lower estimated census sizes (Wiedenfeld 1994). However, the relative estimates of genetic diversity for *A. m. cyanoptera* were higher than the estimate for the Brazilian *A. m. macao* sample, as well as the *A. militaris* and *A. chloropterus* samples (Table 1). Our estimates of divergence, especially after accounting for intra-taxonomic diversity, all gave quantifiably similar results to those of our nuclear phylogenetic and clustering analyses (Table 2).

Our explicit examination of phylogenetic incongruence between South and Central American *A. macao* populations initially suggested higher rates of post-divergence genetic exchange between the Brazilian and Costa Rican *A. m. macao* populations, relative to rates between the Brazilian *A. m. macao* and Central American *A. m. cyanoptera* (Costa Rican and Guatemalan samples combined, Fig. 4). However, when we controlled for geographic distance disparities among the samples by removing the Guatemalan *A. m. cyanoptera*, differences in the number of phylogenetically incongruent sites were was no longer significant (Fig. 4). This result may indicate limited gene flow between South and Central American populations of *A. macao*, a conclusion also drawn from our prior population assessments using mitochondrial haplotype data (Schmidt et al. 2020). Rather, observed incongruences may be the result of incomplete lineage sorting during taxonomic divergence. We found no evidence for ongoing genetic exchange between *A. m*. *macao* and *A. m. cyanoptera* in Costa Rica (Fig. 4), which is also in agreement with our previous analyses (Schmidt et al. 2020).

Taken together, the results presented here suggest that the two subspecies in Central American represent a relatively recent taxonomic divergence, most likely in situ. The unique geographic barriers in Central America may have played a large role in facilitating their formation (e.g., the Cordillera Central of Costa Rica, Figure S1). Presently, there is little evidence from genetic analyses that extensive hybridization occurs between *A. m*. *macao* and *A. m. cyanoptera* in Central America. This absence of hybridization may also be a consequence of these same geographic barriers. Future studies combining both more extensive population-level sampling and nuclear genetic markers will provide better resolution to this question. Additionally, the extent to which divergent natural selection was important in forming unique taxonomic entities within *A. macao* remains an interesting question for further research.

### Electronic supplementary material

Below is the link to the electronic supplementary material.

Supplementary Material 1

Supplementary Material 2

Supplementary Material 3

## Data Availability

The raw sequence reads generated for this study are available from the NCBI SRA database (BioProject: PRJNA997712; BioSample accession numbers: SAMN36685100-SAMN36685106).
