# Supplementary material for: Patterns of cytonuclear discordance and divergence between subspecies of the scarlet macaw (Ara macao) in Central America
Source: Genetica. 2023 Aug 23;151(4-5):281–92. doi: 10.1007/s10709-023-00193-x (PMC10654179; doi:10.1007/s10709-023-00193-x)
Supplement: Supplementary file 1 — Supplementary Material 1 [file 10709_2023_193_MOESM1_ESM.pdf]

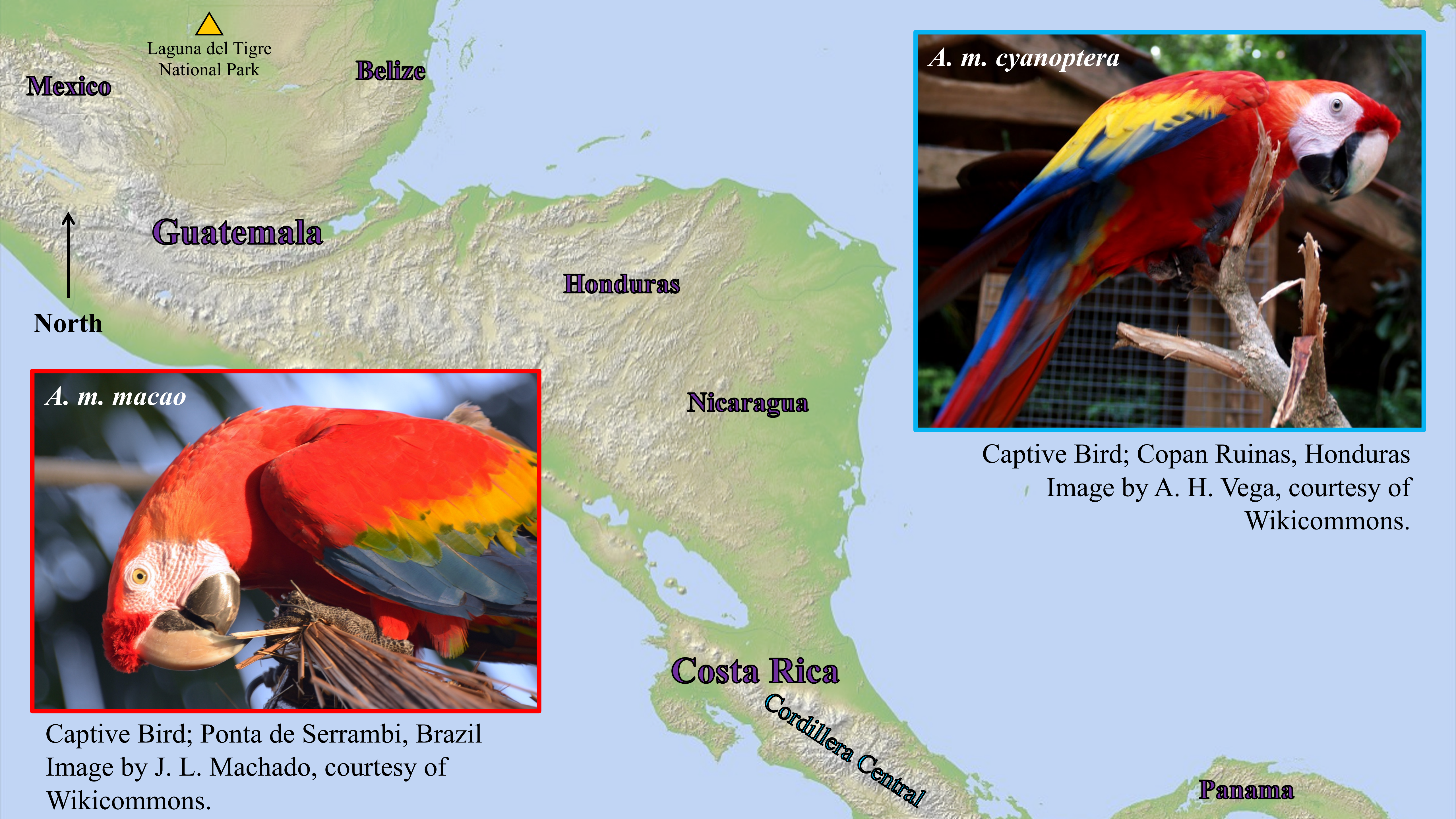

Captive Bird; Copan Ruinas, Honduras
Image by A. H. Vega, courtesy of
Wikicommons.

Captive Bird; Ponta de Serrambi, Brazil
Image by J. L. Machado, courtesy of
Wikicommons.
