# Supplementary material for: Patterns of cytonuclear discordance and divergence between subspecies of the scarlet macaw (Ara macao) in Central America
Source: Genetica. 2023 Aug 23;151(4-5):281–92. doi: 10.1007/s10709-023-00193-x (PMC10654179; doi:10.1007/s10709-023-00193-x)
Supplement: Supplementary file 2 — Supplementary Material 2 [file 10709_2023_193_MOESM2_ESM.pdf]

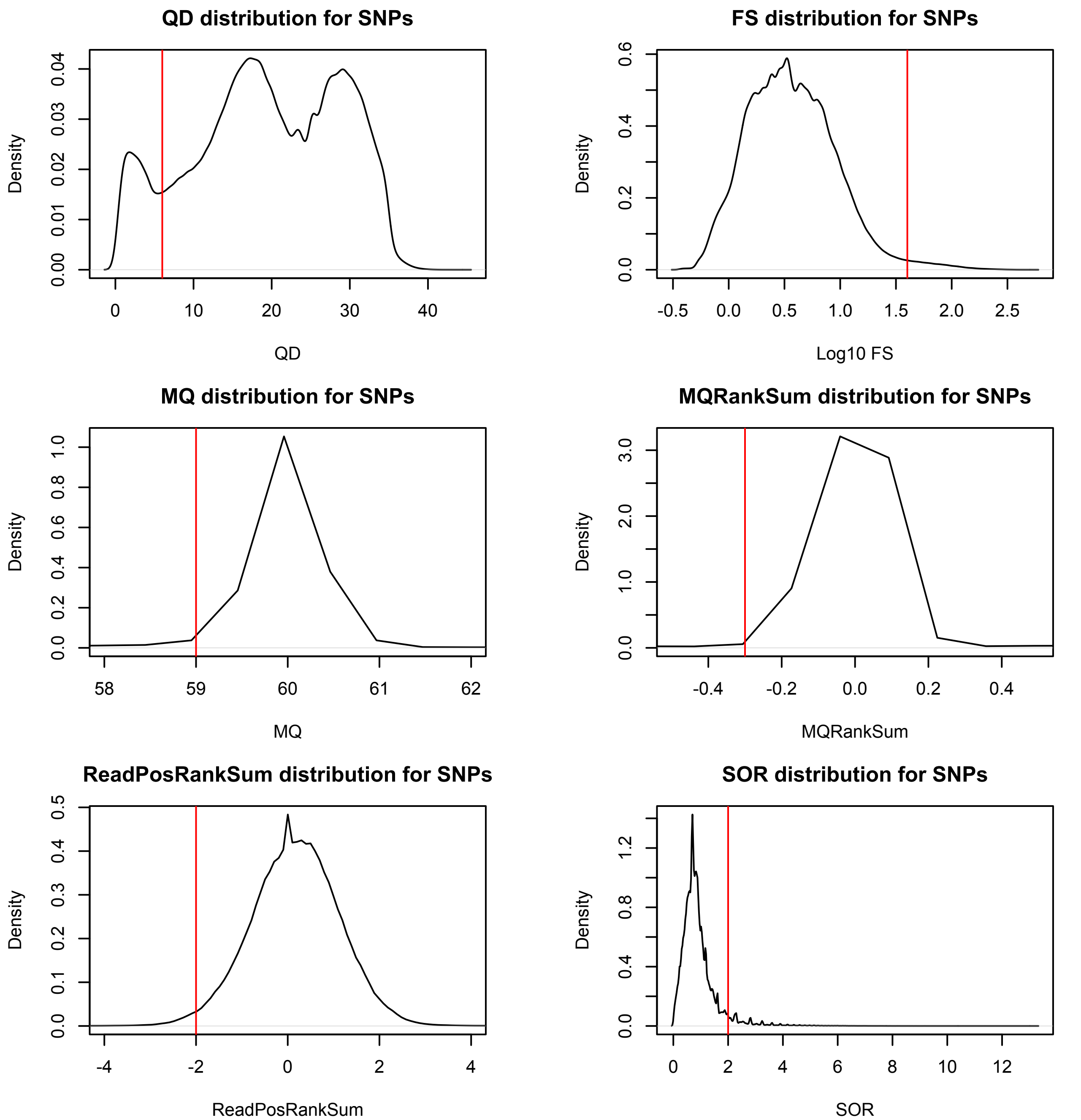
QD distribution for SNPs
Density
0.00 0.01 0.02 0.03 0.04
0 10 20 30 40
QD
FS distribution for SNPs
Density
0.0 0.2 0.4 0.6
-0.5 0.0 0.5 1.0 1.5 2.0 2.5
Log10 FS
MQ distribution for SNPs
Density
0.0 0.2 0.4 0.6 0.8 1.0
58 59 60 61 62
MQ
MQRankSum distribution for SNPs
Density
0.0 1.0 2.0 3.0
-0.4 -0.2 0.0 0.2 0.4
MQRankSum
ReadPosRankSum distribution for SNPs
Density
0.0 0.1 0.2 0.3 0.4 0.5
-4 -2 0 2 4
ReadPosRankSum
SOR distribution for SNPs
Density
0.0 0.4 0.8 1.2
0 2 4 6 8 10 12
SOR
